# Outcomes of Orthopaedic Infections in Recreational Intravenous Drug Users Requiring Long-term Antibiotic Treatment

**DOI:** 10.5435/JAAOSGlobal-D-22-00108

**Published:** 2022-06-10

**Authors:** Erin Stockwell, Kent Rinehart, Emily Boes, Allyson Pietrok, Angela Hewlett, Curtis Hartman, Philipp Streubel

**Affiliations:** From the Department of Orthopaedic Surgery & Rehabilitation, University of Nebraska Medical Center (Dr. Stockwell, Pietrok, and Dr. Hartman); the Indiana Hand to Shoulder Center (Dr. Rinehart); the Department of Orthopaedic Surgery, University of Utah (Dr. Boes); the Department of Internal Medicine, University of Nebraska Medical Center (Dr. Hewlett); and the Cleveland Clinic Florida (Dr. Streubel).

## Abstract

Patients who participate in recreational injection drug use (RIVDU) have an increased risk of orthopaedic infections requiring prolonged treatment with intravenous antibiotics. This study reviews clinical outcomes and complications in RIVDU and have orthopaedic infections requiring long term antibiotic therapy (>4 weeks) and compares these outcomes to non-RIVDU patients. In this retrospective review, patients were divided into cohorts based on RIVDU history; the RIVDU cohort was further divided into subcohorts based on treatment location. Cohorts and subcohorts were compared to evaluate clinical outcomes. Between the two main cohorts, there was a statistically significant difference in treatment compliance (*P* = 0.0012) and no statistically significant differences for infection resolution at 6- or 12-month follow-up, hospital readmission, or mortality. At the 6-month follow-up, RIVDU patients who remained inpatient had 100% resolution of infection, which was significantly better than the resolution of all other cohorts (*P* = 0.0019).

No differences were observed between the remaining subcohorts for resolution of infection by 12 months, catheter complications, or loss to follow-up. Our findings demonstrate an increased rate of failure in outpatient parenteral antibiotic therapy in RIVDU patients, and this population has better clinical outcomes when they remain inpatient for the duration of treatment.

Recreational intravenous (IV) drug use (RIVDU) is prevalent in society with an estimated 13 million people using injectable drugs globally.^1^ People who inject drugs have an increased risk of premature mortality from drug overdose, suicide, and complications of HIV.^2-4^ In addition, this demographic has an increased prevalence of many types of infections including hepatitis, skin and soft-tissue infections, and bone and joint infections.^5,6^ Osteomyelitis, prosthetic joint infections, septic arthritis, and epidural abscesses commonly occur in this population and with an increased risk of clinical complications; the treatment of these conditions often requires prolonged antibiotic treatment in addition to surgical intervention.^7,8,9,10,11^

Antibiotic treatments are frequently administered through central venous catheters to optimize drug bioavailability. Prolonged courses of IV antibiotic therapy are traditionally administered in an outpatient setting given the safety, efficacy profile, and markedly reduced cost compared with inpatient administration.^12^ Although this can be achieved with daily appointments at outpatient infusion centers, cost and convenience are further improved with in-home medication administration.^12^

In the RIVDU population, outpatient parenteral antibiotic therapy (OPAT) has historically been shown to be associated with high rates of failure.^12,13^ In this subset of patients, direct IV access can lead to increased drug use with associated complications such as endocarditis and sepsis.^14,15^ Although high rates of failure of OPAT have been documented in the RIVDU population, the published literature consists of only small case series.^9,13^ An increased interest in this clinically challenging scenario has prompted additional investigation into risks and outcomes of OPAT in this high-risk population, including one small series directly comparing outcomes between patients with and without a history of drug use and a large retrospective review of outcomes of RIVDU and non-RIVDU patients with additional stratification based on housing status.^16-18^ Ultimately, given the lack of available data on the topic, the most recent guidelines from the Infectious Diseases Society of America do not give a formal recommendation regarding the use of OPAT in RIVDU.^9^ The available data specifically addressing OPAT in RIVDU with orthopaedic infections is even more scarce.

The goal of this study was to review clinical outcomes and complications in RIVDU patients with orthopaedic infections who required long-term antibiotic therapy (>4 weeks) and to compare these outcomes to non-RIVDU patients. The primary outcome measures between the RIVDU and non-RIVDU groups were treatment compliance and infection resolution; secondary outcome measures included hospital readmission and mortality. Additional outcome measures within the RIVDU subcohorts included infection resolution, hospital length of stay, hospital readmission rates, and catheter complications. We hypothesized that the non-RIVDU cohort would have higher rates of infection resolution and lower rates of mortality.

## Methods

**Figure 1:**
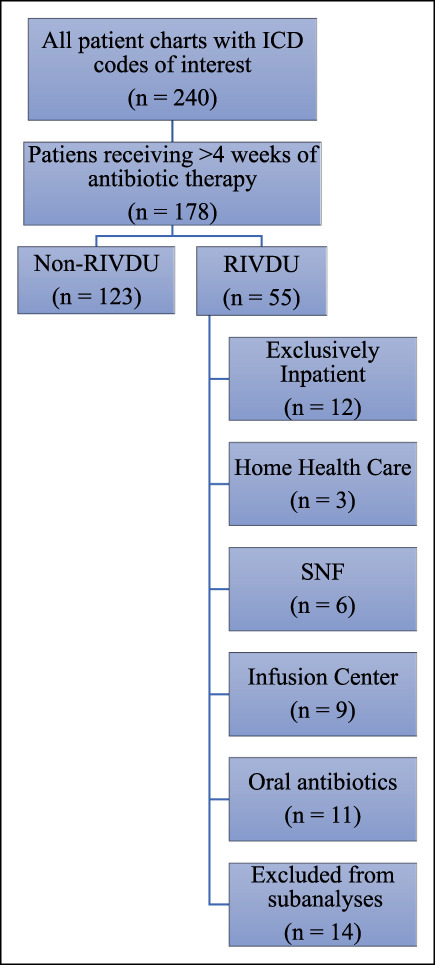
Flowchart showing an overview of study subject inclusion and stratification

After obtaining approval from the Institutional Review Board, a retrospective chart review was conducted on patients admitted to a single academic medical center between August 12, 2012, and December 31, 2018. The starting date for data collection was chosen based on the initiation of an electronic medical record with searchable patient records. This institution serves as a regional referral center serving a population of nearly 1.5 million people. Inclusion criteria were (1) age 18 years or older, (2) patients with a diagnosis of an infection of interest (epidural abscess, osteomyelitis, prosthetic joint infection, and septic arthritis) identified through the electronic medical record based on International Classification of Diseases codes (Supplemental Table 1, http://links.lww.com/JG9/A225), and (3) antibiotic treatment of at least 4 weeks duration. Patients were then further divided into RIVDU and non-RIVDU groups. Patients were excluded from subcohort analyses if they were transferred to an outside facility for further management, were discharged to a correctional facility, underwent acute management with amputation or if they died during hospitalization (Figure 1).

Descriptive statistics were used to summarize variables of interest between the groups including demographics, infection type, tobacco use, and a concomitant diagnosis of diabetes. The Fisher exact test was used to look at associations of categorical variables with RIVDU status including infection resolution, compliance with treatment, hospital readmission rates, catheter complications, and mortality. Infection resolution was defined as the resolution of all symptoms with no indication for additional antibiotic therapy. Treatment compliance was defined as adherence to the prescribed dosing and duration of antibiotic therapy. Hospital readmission was defined as any readmission after discharge related to the orthopaedic infection of interest between diagnosis and infection resolution. Catheter complications were defined as catheter infection, adverse reaction resulting in removal, or abuse of catheter for recreational drug administration. Mortality for each patient was assessed at the time of data collection. The independent sample Student *t*-test or ANOVA was used to compare continuous data between the RIVDU and non-RIVDU cohorts and subcohorts.

Within the RIVDU group, five subcohorts were established based on the location and type of antibiotic administration: exclusively inpatient IV administration, home IV administration by home health care, IV administration at a skilled nursing facility, outpatient IV administration at an infusion center, and home with oral antibiotics (Figure 1). These five subcohorts were compared by evaluating mean hospital length of stay (LOS), resolution of infection at 6 and 12 months, compliance with therapy, hospital readmission rates, and catheter complications. The Fisher exact test was used to look at associations of categorical variables with treatment outcomes. The Kruskal-Wallis test was used to compare the median LOS between the groups; pairwise comparisons of LOS were adjusted using the Dunn test.

All analyses were conducted using SAS, Version 9.4; *P* < 0.05 was considered statistically significant. This study design is consistent with level III evidence.

## Results

A total of 178 patients (123 non-RIVDU and 55 RIVDU) were included in the analysis. An overview of patient characteristics and demographics within each cohort are listed in Table 1. Within the non-RIVDU cohort, 83 and 75 patients had documented follow-up at 6 and 12 months, respectively. Within the RIVDU cohort, 35 and 32 patients had documented follow-up at 6 and 12 months, respectively. No statistically significant differences were observed between the non-RIVDU and RIVDU patient groups for sex, BMI, race/ethnicity, history of diabetes, or infection type. However, a statistically significant difference was observed between the two for tobacco use and mean age; only 29% of the non-RIVDU patients were active tobacco users compared with 70% of the RIVDU patients (*P* < 0.0001), and mean age for non-RIVDU patients was 58 years compared with 47 years for RIVDU patients (*P* < 0.0001). Within the RIVDU cohort, 62% of the patients reported drug use within 30 days and an additional 11% within 12 months of diagnosis of an orthopaedic infection of interest.

**Table 1:** Patient Characteristics

Factor	Non-RIVDU (n = 123)	RIVDU (n = 55)	*P*
Sex			0.8601
Male	87 (70.7%)	38 (69.1%)	
Female	36 (29.3%)	17 (30.9%)	
Age (yr)			**<0.0001**
Mean	57.6	46.8	
Median	60	47	
Range	21-92	27-71	
BMI			0.76
Mean	29.6	29.2	
Median	28.5	27.7	
Range	15.5-61.6	17.2-51.2	
Race/ethnicity			0.8413
White	90 (73.2%)	43 (78.2%)	
Hispanic/Latino	8 (6.5%)	3 (5.5%)	
African American	25 (20.3%)	9 (16.3%)	
History of diabetes			0.6158
Yes	48 (39.7%)	19 (34.6%)	
No	73 (60.3%)	36 (65.4%)	
Tobacco use			**<0.0001**
Current	36 (29.3%)	39 (70.9%)	
Former	36 (29.3%)	11 (20%)	
None	51 (41.4%)	5 (9.1%)	
Infection type			0.1101
Epidural abscess	11 (8.9%)	9 (16.4%)	
Osteomyelitis	58 (47.1%)	26 (47.3%)	
Prosthetic joint infection	27 (22%)	5 (9.1%)	
Septic arthritis	27 (22%)	15 (27.3%)	

RIVDU = recreational injection drug use

A statistically significant difference was observed in patient compliance with IV antibiotic therapy between the two cohorts (*P* = 0.0012) with compliance documented in 85.1% of the non-RIVDU patients and only 61.5% of the RIVDU patients. Contrarily, statistically significant differences were observed between the two cohorts for infection resolution at the 6- or 12-month follow-up after completion of therapy, hospital readmission, or mortality at the time of data collection (Table 2).

**Table 2:** Clinical Outcomes

Factor	Non-RIVDU	RIVDU	*P*
Infection resolution			
6 mo	57/83 (69%)	22/35 (63%)	0.6687
12 mo	60/75 (80%)	10/32 (31%)	0.2212
Therapy compliance	n = 114	n = 52	**0.0012**
Yes	97 (85%)	32 (62%)	
No	17 (15%)	20 (38%)	
Hospital readmission	n = 120	n = 53	
Yes	56 (47%)	28 (53%)	0.5107
No	64 (53%)	25 (47%)	
Catheter complications	n = 67	n = 23	**0.0158**
Yes	4 (6%)	6 (26%)	
No	63 (94%)	17 (74%)	
Mortality	n = 123	n = 54	0.6887
Yes	24 (20%)	12 (22%)	
No	99 (80%)	42 (78%)	

RIVDU = recreational injection drug use

A total of 41 of the 55 RIVDU patients were included in additional RIVDU subcohort analyses. Reasons for exclusions from subcohort analyses included transfer to an outside facility for additional management (n = 1), discharge to a correctional facility (n = 3), acute management with amputation (n = 8), and expiration during hospitalization (n = 2). Those who remained admitted to the hospital for the duration of therapy had a median LOS of 41 days, which was significantly longer than all other cohorts (*P* < 0.0001). At the 6-month follow-up, this subcohort also had 100% resolution of infection, which was significantly better than the resolution of all other subcohorts (*P* = 0.0019). The inpatient cohort also had lower readmission rates throughout the 7-year study period compared with the other cohorts (*P* = 0.0130). One patient in the inpatient subcohort was discharged against medical advice for less than 24 hours, readmitted for continued treatment, and classified as a hospital readmission. No difference was observed between the five patient cohorts for resolution of infection at the 12-month follow-up or catheter complications (Table 3).

**Table 3:** Recreational Injection Drug Use Subcohort Analyses

Factor	Hospital, n = 12	SNF, n = 6	Infusion Center, n = 9	Home with HHC, n = 3	Home, PO, n = 11	*P*
Length of stay (d)						**<0.0001**
Mean	42.33	18.8	11.7	7.7	7.7	
Median	41	17	6	8	5	
Range	28-57	10-36	1-28	7-8	2-28	
Infection resolution at 6 mo						**0.0194**
Yes	8	2	6	3	0	
No	0	2	2	0	2	
Therapy compliance						**0.0058**
Yes	10	0	6	3	2	
No	2	4	3	0	5	
Hospital readmission						**0.0130**
Yes	1	5	4	0	4	
No	11	1	5	3	5	
Catheter complications						0.1390
Yes	0	1	0	0	1	
No	12	5	9	3	3	

HHC = home health care, SNF = skilled nursing facility

## Discussion

Treatment of orthopaedic infections in RIVDU patients remains a challenging problem. Although recent data suggest that prolonged oral antibiotic treatment may offer comparable outcomes for orthopaedic infections, even for nonquinolone antibiotics, IV antibiotic therapy is often still used for the treatment of these infections.^4,5,8,9^ In many instances, IV antibiotic therapy can be safely and effectively delivered in the outpatient setting using an indwelling catheter.^9,12,13^ This route of administration is however often fraught with possible complications in RIVDU patients.^5,13^ Possible complications include difficulty obtaining IV access, possible endocarditis and other blood-borne infections, and catheter site infections. Several factors may influence the ability to adequately treat these infections. Factors related to the host play a key role because patients may be malnourished, be immunocompromised, and be less likely to comply with recommended treatment.

This study sought to compare clinical outcomes and complications between RIVDU and non-RIVDU patients with orthopaedic infections that required long-term antibiotic therapy. In agreement with our hypothesis, our results demonstrate that there is a markedly higher rate of compliance with antibiotic therapy in the non-RIVDU group. Interestingly, against the prediction of our hypothesis, this did not correlate with a statistically significant difference between rates of infection resolution between the two cohorts at the 6- or 12-month follow-up. The lack of a notable difference between hospital readmission rates between the two groups reinforces the notion that these types of orthopaedic infections are often difficult to treat in all populations.

Similar to previous studies, our findings demonstrate a statistically significant increased rate of failure in OPAT therapy in the RIVDU group.^9,12,13,17^ Consistent with findings by Ciccarone et al demonstrating an increased rate of clinical complications such as endocarditis and sepsis in RIVDU patients with prolonged, direct IV access, our study found a statistically significant increase in catheter-associated complications in the RIVDU cohort compared with non-RIVDU patients.^14,15^

Recent efforts have been made to directly evaluate outcomes in RIVDU and non-RIVDU populations. Appa et al^16^ directly compared OPAT outcomes between patients with and without a history of drug abuse and found no differences in therapy completion or hospital readmission rates between the two groups. Although encouraging, this study did not specifically evaluate infection resolution as an outcome and patients with active drug use were excluded. In a direct comparison between RIVDU and non-RIVDU cohorts, our study fails to demonstrate a statistically significant difference in infection resolution at 6- and 12-month follow-ups. However, comparisons were made in fairly small subgroups with a possibility to be underpowered to show that such differences actually exist.

Assessments to stratify risk in patients with a history of RIVDU have been developed.^19^ Some centers also use integrated care models to assess and manage patients with a history of RIVDU, which may include the provision of counseling and pharmacotherapy on an inpatient and outpatient basis.^20^ These multidisciplinary teams may include addiction psychiatry, OPAT pharmacists, infectious disease specialists, and primary care teams and provide a comprehensive approach to the management of patients with a history of RIVDU who require IV antibiotic therapy.

For non-RIVDU patients, great efforts have been taken to streamline OPAT for the treatment of infections requiring prolonged IV antibiotic therapy. Administration of IV antibiotics often offers greater drug bioavailability, and utilization of therapy in an outpatient setting has been demonstrated to be safe and less expensive.^12^ Within our RIVDU subcohort analyses, infection resolution at the 6-month follow-up was markedly higher only for patients who admitted for the duration of their IV antibiotic therapy. However, although statistical significance was not achieved, those discharged home with home health care had a 100% infection resolution at the 6-month follow up, 100% therapy compliance, no hospital readmissions, and no catheter complications. Larger subcohort numbers could result in statistically significant findings. This could have a drastic effect on future standards of care for the treatment of orthopaedic infections in this patient population with potentially improved outcomes and markedly reduced healthcare costs.

Recent data suggest that oral antibiotic treatment may yield similar outcomes to IV administration for the treatment of bone and joint infections.^21^ If such results could be confirmed in the RIVDU population, catheter-related complication rates could be potentially avoided. However, as shown by our study, resolution was markedly lower after oral antibiotics therapy; additional studies on the cause of this are warranted. Factors such as organism virulence and poor compliance could play a role.

This study has several limitations. The retrospective design inherently results in a lack of comprehensive data collection due to heterogeneity in medical record documentation. Both the patient population of interest and the lack of provider-requested follow-up beyond eradication of an infection also limit the available outcome data at 6- and 12-month follow-ups. Ideally, a future prospective study comparing non-RIVDU and RIVDU cohorts would be conducted to validate our retrospective findings.

This study did not account for other possible confounding variables such as microorganism typification or clinical complexity. Furthermore, possible malnutrition and immune compromise can play an important role in a host's ability to clear an infection, and no analysis related to these variables was conducted. It is theoretically possible that inpatient treatment may have had an effect not only in optimizing IV antibiotic administration but also to reduce the exposure to tobacco and improve nutritional status.

Patients with a history of RIVDU who suffer from bone or joint infections have not been given adequate attention in the orthopaedic or infectious disease literature. More research is needed to determine how this patient population can achieve equivalent clinical outcomes while still discharging from the hospital in a timely manner. Future efforts should be aimed at designing a prospective study comparing the outcomes of RIVDU patients in different treatment scenarios. This would require active participation of orthopaedic surgeons, infectious disease specialists, home health care, and skilled nursing facilities.
